# Arginine- and Polyamine-Induced Lactic Acid Resistance in *Neisseria gonorrhoeae*

**DOI:** 10.1371/journal.pone.0147637

**Published:** 2016-01-25

**Authors:** Zheng Gong, M. Matt Tang, Xueliang Wu, Nancy Phillips, Dariusz Galkowski, Gary A. Jarvis, Huizhou Fan

**Affiliations:** 1 Department of Pharmacology, Rutgers University Robert Wood Johnson Medical School, Piscataway, New Jersey, United States of America; 2 Department of Urology, Hunan University of Traditional Chinese Medicine-affiliated Ningxiang People’s Hospital, Changsha, Hunan, China; 3 Department of Obstetrics, Gynecology and Reproductive Sciences, and Women’s Health Institute, Rutgers University, New Brunswick, New Jersey, United States of America; 4 Public Health and Environmental Laboratories, New Jersey Department of Health, Ewing, New Jersey, United States of America; 5 Department of Pathology and Laboratory Medicine, Rutgers University Robert Wood Johnson Medical School, Piscataway, New Jersey, United States of America; 6 Center for Immunochemistry, Veterans Affairs Medical Center, San Francisco, California, United States of America; 7 Department of Laboratory Medicine, University of California, San Francisco, California 94143, United States of America; Univ. of Texas Health Science Center at San Antonio, UNITED STATES

## Abstract

Microbe-derived lactic acid protects women from pathogens in their genital tract. The purpose of this study was to determine lactic acid susceptibility of *Neisseria gonorrhoeae*, and identify potential acid resistance mechanisms present in this pathogen. Tested *in vitro*, lactic acid killed all 10 gonococcal strains analyzed in a low pH-dependent manner. Full inactivation occurred at pH 4.5. At low pH, lactic acid treatment resulted in the entry of the DNA-binding fluorochrome propidium iodide into the microbial cells, suggesting that hydrogen ions from lactic acid compromise the integrity of the bacterial cell wall/membrane. Most likely, hydrogen ions also inactivate intracellular proteins since arginine rendered significant protection against lactic acid presumably through action of the gonococcal arginine decarboxylase, an enzyme located in the bacterial cytoplasm. Surprisingly, arginine also lessened lactic acid-mediated cell wall/membrane disruption. This effect is probably mediated by agmatine, a triamine product of arginine decarboxylase, since agmatine demonstrated a stronger protective effect on GC than arginine at equal molar concentration. In addition to agmatine, diamines cadaverine and putrescine, which are generated by bacterial vaginosis-associated microbes, also induced significant resistance to lactic acid-mediated GC killing and cell wall/membrane disruption. These findings suggest that the arginine-rich semen protects gonococci through both neutralization-dependent and independent mechanisms, whereas polyamine-induced acid resistance contributes to the increased risk of gonorrhea in women with bacterial vaginosis.

## Introduction

Among the 2.3 million notifiable infections reported to the Center for Disease Control and Prevention (CDC) of the United States, two of every three were sexually transmitted infections (STI) [1]. Yet, CDC estimates this reported figure to be only one tenth of the actual number of new STI infections. Annual health care costs for treating STI and related complications are estimated to be $16 billion [2].

One of the most common STI is gonorrhea, caused by *Neisseria gonorrhoeae*, also referred as gonococcus (GC). In the United States, CDC estimates that more than 800,000 new gonorrheal cases occur each year. There were three consecutive years of increases in reported gonorrheal cases from 2010 and 2012. Although the number of reported cases decreased slightly in 2013, it increased by 5% in 2014, and reached the highest level since 2008 [3]. Gonorrhea in women often exhibits mild or even no symptoms. However, if left untreated, the infection can lead to serious complications including pelvic inflammatory symptoms, ectopic pregnancy, and tubal factor infertility [4]. In addition, gonorrhea is known as a cofactor for HIV/AIDS [5–7]. Alarmingly, drug-resistant GC strains are widespread. As a result, currently effective treatment of gonorrhea requires multiple antibiotics [8].

Most reproductive-age women have an acidic vaginal environment owing to the overwhelmingly large numbers of lactic acid-producing bacteria, mainly lactobacilli, present in the vagina [9–11]. However, in women with bacterial vaginosis, lactobacilli are replaced by other bacteria, leading to increased vaginal pH (>4.5) [11–14]. A number of population-based studies have associated bacterial vaginosis with increased risks of STIs including gonorrhea, and therefore, have suggested a protective role of lactic acid-generated acidity in defense against STI [7, 10, 15–20]. However, to the best of our knowledge, the effect of lactic acid on GC survival has not been experimentally documented to date. Although HCl-mediated GC killing has been reported [21, 22], studies have demonstrated that lactic acid and HCl differ in their antimicrobial activities [23, 24]. We have recently determined the lactic acid susceptibility of *Chlamydia trachomatis*, another major sexually transmitted bacterial pathogen [23]. In this report, we show that multiple GC strains are fully inactivated by lactic acid at pH 4.5. Interestingly, the microbicidal activity of lactic acid is significantly weakened in the presence of arginine and polyamines. Our findings indicate that semen, known to be rich in arginine [25], helps GC survive and colonize the female genital tract by both neutralizing lactic acid and inducing arginine-dependent acid resistance. In addition, our observations suggest that both reduced lactic acid production and increased polyamines production associated with bacterial vaginosis increase the risk for gonorrhea.

## Materials and Methods

### Media

GC liquid medium (GCL) contained (per liter) 15 g proteose peptone 3 (EMD Millipore), 1 g soluble starch, 4.0 g K_2_HPO_4_, 1.0 g KH_2_PO_4_, 5 g NaCl, 0.4 g glucose, 10 mg glutamine, 20 μg cocarboxylase and 0.5 mg Fe(NO_3_)_3_•9H_2_O. GCL-LA was prepared by supplementing GCL with 100 mM lactic acid, and adjusted to pH 4.0, 4.5, 5.0, 5.5 and 6.0 using NaOH. NEG buffer (pH 7.0) contained 72 mM NaHPO_4_, 5.5 mM (NH_4_)H_2_PO_4_, 10 mM KCl, 0.25 mM MgSO_4_, 0.1 mM CaCl_2_, 28 mM dextrose and 9.5 mM sodium citrate, and was adjusted to pH 4.9, 5.0 or 5.2 using lactic acid. NEG-LA was prepared by supplementing NEG with 75 mM lactic acid, and adjusted to desired pH using NaOH. Proteose peptone 3 was purchased from EMD Millipore. All other medium components were purchased from Sigma.

### Strain information

GC ATCC 19424 was purchased from ATCC. FA1090 was provided by Dr. Ann E. Jerse [26]. FA5100T1, MS11MkCT1, FA1342T1, Can116 and KGC654 were from the collection of the Jarvis Laboratory. NJ1, NJ2 and NJ3 were isolated by the Public Health and Environmental Laboratories of New Jersey.

### Preparation of GC stocks

Bacteria in well-separated colonies that formed on GC Agar plates after 17–20 h incubation at 37°C were harvested in 0.9% NaCl (saline) with the aid of a 1.5 cm (diameter) aluminum loop. A 100 μl sample of the GC suspension was mixed with 900 μl GCL. Optical density at 600 nm (OD_600_) for the resulting diluted bacterial suspension was measured.

### GC killing tests

On ice, 10 μl of GC saline suspension were diluted with saline to 0.005 OD_600_. On 96-well plates, 11 μl of the diluted suspension was mixed with 100 μl GCL-LA or control GCL (pH 7.0). The plates were placed in a 37°C incubator containing air supplemented with 5% CO_2_. 40 min later, the bacteria were subjected to 10 fold serial dilution using GCL, and plated onto GC Agar plates. In experiments determining the effects of specific amino acids or polyamine on lactic acid-mediated GC killing, GCL-LA was substituted with the NEG-LA. Direct alkylating or acidifying effects of amino acids and polyamines on the medium were eliminated by readjusting pH using additional lactic acid or NaOH as needed. All killing experiments were performed in triplicate.

### Propidium iodide (PI) staining

Bacteria were harvested in saline. Samples of the bacterial suspension were diluted to 1.0 OD_600_ using no less than nine volumes of GCL-LA, NEG-LA or NEG-LA containing arginine, glutamic acid, lysine, agmatine, cadaverine or putrescine (all purchased from Sigma) at indicated pH. The final volume for each treatment was 1 ml. After 40 min incubation in a 37°C incubator containing air supplemented with 5% CO_2_, bacteria were collected by centrifugation and resuspended in phosphate-buffered saline containing 0.1% gelatin and 10 ng/ml PI. The bacterial suspension was incubated at room temperature for 10 min. Bacteria were again centrifuged, and washed once with the buffer supplemented with only 0.1% gelatin. Smears were prepared and viewed with a 100 X oil-immersion objective under an Olympus IX51 fluorescence microscope. All fluorescence images were acquired with equal exposure time for the same experiment.

## Results

### Lactic acid kills GC in a low pH-dependent manner

**Fig 1:**
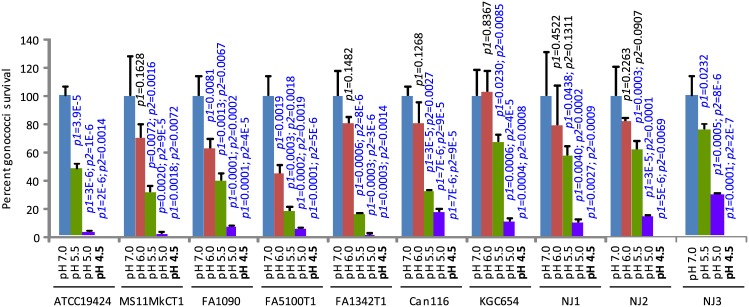
Lactic acid-mediated, pH-dependent GC killing. The GCL medium was supplemented with 100 mM lactic acid and adjusted to indicated pH using NaOH. The resulting GCL-LA and control lactic acid-free medium (pH 7.0) were used to treat GC. Surviving bacteria were quantified by plating the treatments onto GC Agar plates. Number of input bacteria per assay were 10,000–70,000 colony-forming units (CFU), as determined by treatment with control GCL. Note that treatment with pH 4.5 (shown in boldface) resulted in complete killing in all GC strains. Data represent averages ± standard deviations of triplicate experiments. *p-*values for difference in numbers of colony-forming units between samples treated with GCL-LA and control GCL (pH 7.0) were calculated using *t* tests (two-tailed between control and pH 6.0, and one-tailed for all other comparisons). *p1* is the *p-*values for difference between samples treated with GCL-LA and control GCL (pH 7.0), whereas *p2* is the difference between samples treated with GCL-LA at the pH that the *p-*value appears above, and samples treated with GCL-LA that has a 0.5 unit higher pH. *p-*values of < 0.05, which indicate statistically significant differences, are shown in blue font, while those of > 0.05 are shown in black font.

The lactic acid concentration in the vaginal lumen of women with a *Lactobacillus-*dominating microbiota ranges from 88 to 165 mM (average: 110 mM) [11]. To determine the effect of a lactic acid-generated low pH environment on gonorrheal pathogenesis, we compared bacterial survival in the control GCL medium (pH 7.0) and GCL-LA (GCL supplemented with 100 mM lactic acid) adjusted to pH 4.0, 4.5, 5.0, 5.5 and 6.0 using NaOH. GCL-LA exhibited a microbicidal effect on all 10 GC strains tested in a low pH-dependent manner (Fig 1). Accordingly, incubation at pH 4.0 (data not shown) and 4.5 resulted in complete loss of colony-forming activity; in sharp contrast, incubation at pH 6.0 resulted in poor or even lack of GC killing. Between pH 4.5 and 5.5, every increase of 0.5 pH unit resulted in a statistically significantly lower killing efficiency for all strains. Increase of pH from 5.5 to 6.0 was also accompanied by a significantly lesser killing for all but 2 strains, NJ1 and NJ2. Even for the latter two strains, decreased killing was observed when pH was raised from 5.5 to 6.0 although the *p-*values only trended toward significance in triplicate experiments (Fig 1).

### Lactic acid treatment causes propidium iodide (PI) to enter GC

**Fig 2:**
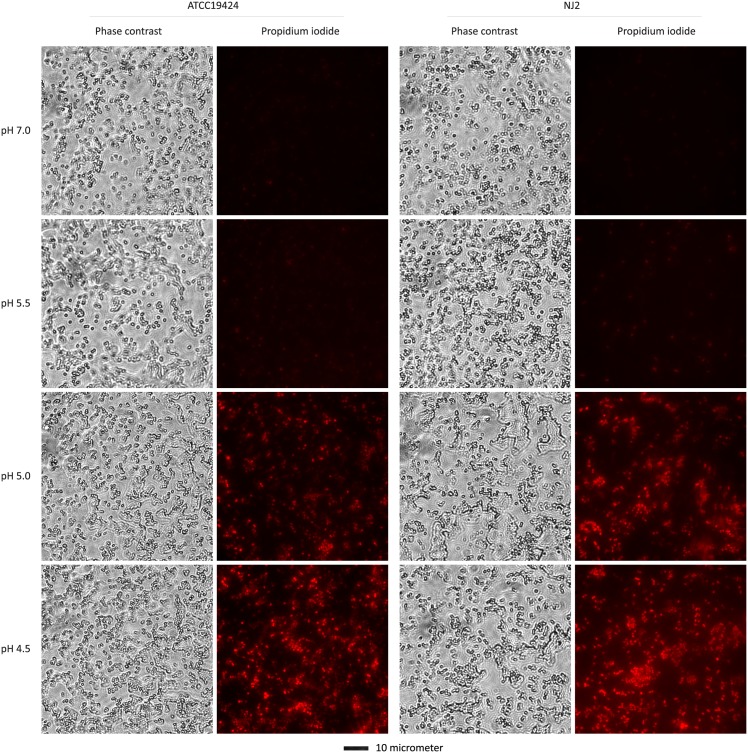
Lactic acid-mediated, pH-dependent entry of propidium iodide (PI) into GC cells. The membrane-impermeable fluorescent molecule PI was added to GC after the bacteria were treated with GCL-LA adjusted to indicated pH. After a wash to remove the free fluorochrome, smears were prepared and images were acquired. A scale bar is at the bottom of the figure.

We used the DNA-binding fluorochrome PI to determine the mechanism underlying GC killing by lactic acid using two bacterial strains, ATCC19424 and NJ2. As expected, the membrane impermeable molecule is largely excluded from the cells of both strains treated with GCL-LA adjusted to pH 7.0 (Fig 2). However, apparent staining with the fluorochrome was observed in bacteria treated with GCL-LA starting at pH 5.5, which became increasingly prominent as the pH decreased to 5.0 and 4.5 (Fig 2). These findings suggest that lactic acid-mediated low pH kills GC by compromising the integrity of the bacterial cell wall/membrane.

### Arginine decreases lactic acid-mediated GC killing

**Fig 3:**
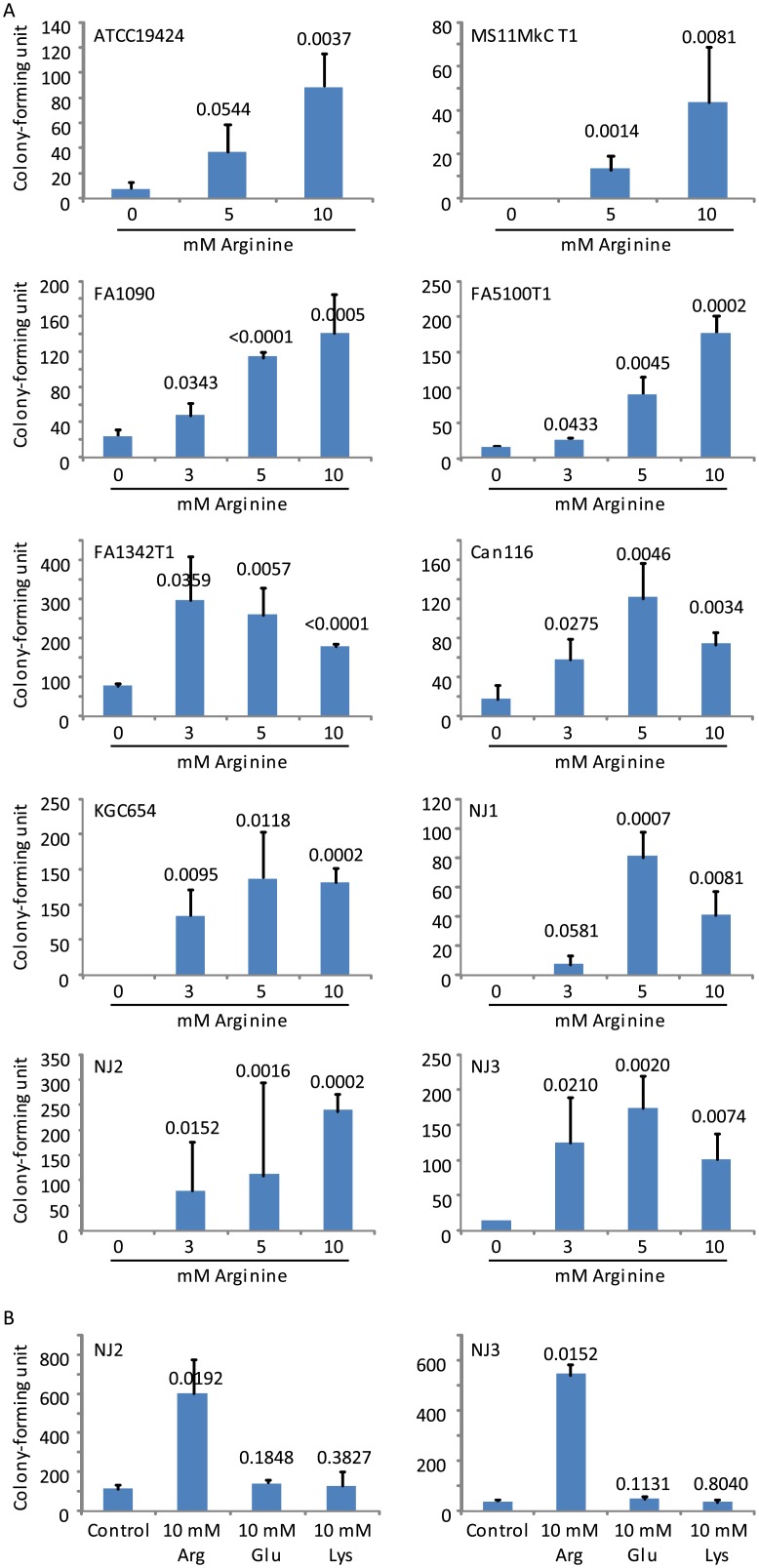
Arginine-induced acid resistance in GC. Bacteria were treated with the defined medium NEG supplemented with 75 mM lactic acid (NEG-LA) with or without further supplementation with arginine at the indicated concentration (A), or with arginine, glutamic acid or lysine at 10 mM (B). All media were adjusted to pH 5.0. Numbers of surviving bacteria were determined by plating the treatments onto GC Agar plates. Data represent averages ± standard deviations of triplicate experiments. Numbers above the bars were *p-*values (one-tailed *t* tests) for difference in surviving bacteria between samples treated with the control NEG-LA (pH 5.0) and those treated with NEG-LA supplemented with an indicated concentration of arginine (A), glutamate or lysine (B). Number of input bacteria per assay were 10,000–40,000 CFU, as determined by treatment with control NEG-LA (pH 7.0).

Arginine, glutamic acid and lysine increase the resistance of *E*. *coli* and other bacteria to the microbicidal activity of low pH generated by HCl [27, 28]. To determine whether similar acid resistance mechanisms exist in GC in response to lactic acid, we substituted the rich GCL medium with NEG, an amino acid-free medium, for acid killing experiments. As expected, lactic acid also demonstrated low pH-dependent GC-killing activity in NEG. Interestingly, 9 of the 10 GC strains showed highly significantly improved survival in the presence of arginine at both 10 mM and 5 mM, whereas the *p* value for ATCC19424 treated with 10 mM arginine was also highly significant (0.0037), and with 5 mM arginine trended toward significance at 0.0544 (Fig 3A). Eight strains were also tested with 3 mM arginine, and all demonstrated increased survival although the *p* value for NJ1 trended toward significance at 0.0581 (Fig 3A). In contrast to 10 mM arginine, neither 10 mM glutamic acid nor 10 mM lysine showed any effect on bacterial survival when tested concurrently with arginine against strains NJ2 and NJ3 (Fig 3B). Taken together, our data suggest that arginine but not glutamic acid or lysine is effective in inducing acid resistance in GC.

### Arginine reduces lactic acid-mediated PI entry

**Fig 4:**
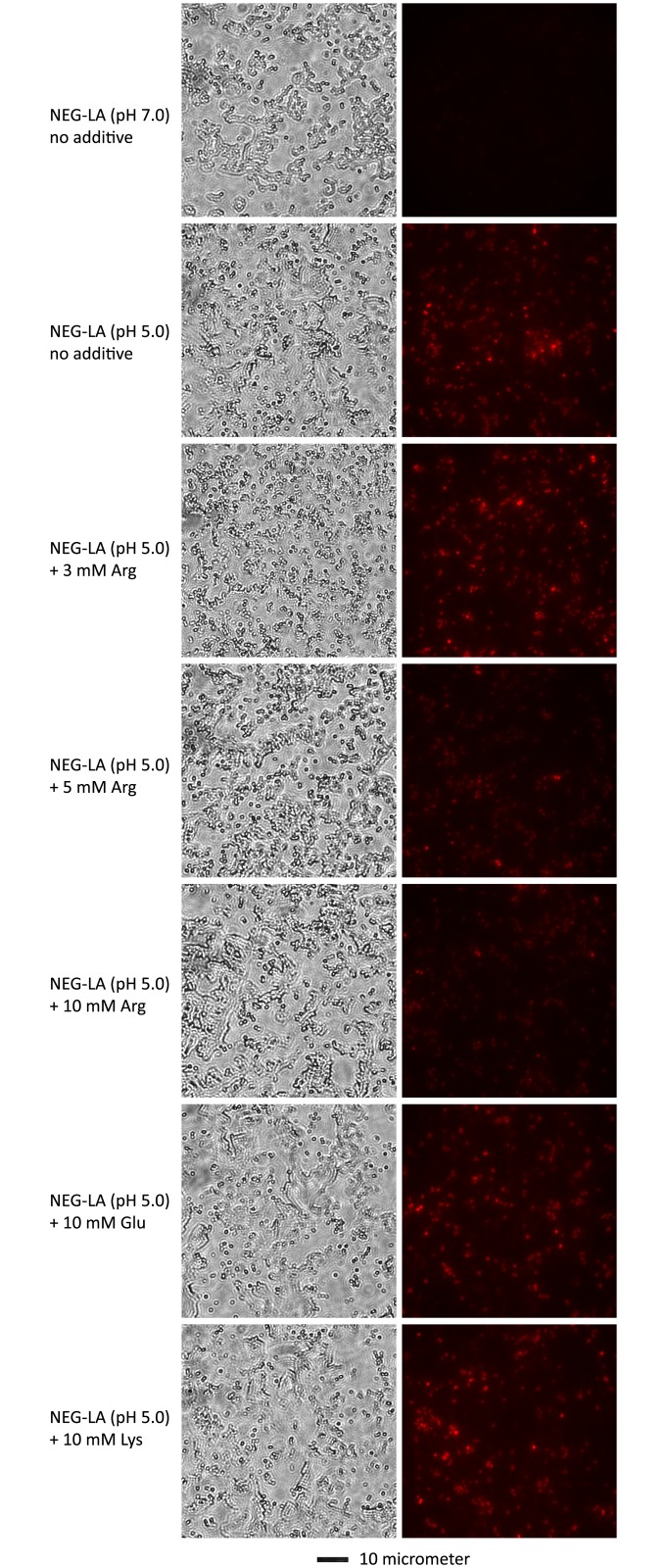
Arginine-, but not glutamate- or lysine-mediated inhibition of lactic acid-induced wall/membrane permeation. NJ2 were first treated with NEG-LA (pH 5.0) or NEG-LA containing indicated supplements as described in the legend to Fig 3 legend, then exposed to PI, and finally imaged with a fluorescent microscope (Fig 2). Matching phase contrast images are presented on the left. A scale bar is at the bottom.

Arginine causes acid resistance through a hydrogen ion-consuming reaction catalyzed by the arginine decarboxylase in the bacterial cytoplasm [27, 28]. Thus, the demonstration of arginine-dependent lactic acid resistance in GC (Fig 3) suggests that hydrogen ions also cause inactivation of intracellular molecules, in addition to disruption of cell wall/membrane as demonstrated in Fig 2. Surprisingly, compared to GC treated with control NEG-LA (pH 5.0), bacteria treated with the same medium supplemented with either 10 or 5 mM arginine displayed noticeably decreased PI staining although the effect of 3 mM was not obvious (Fig 4). In comparison, neither 10 mM glutamic acid nor 10 mM lysine had an apparent effect on PI entry. These results suggest that arginine not only serves to decrease the intracellular hydrogen ion concentration, but also can ameliorate lactic acid-induced cell wall/membrane disruption.

### Agmatine lessens lactic acid-induced GC killing and PI entry

**Fig 5:**
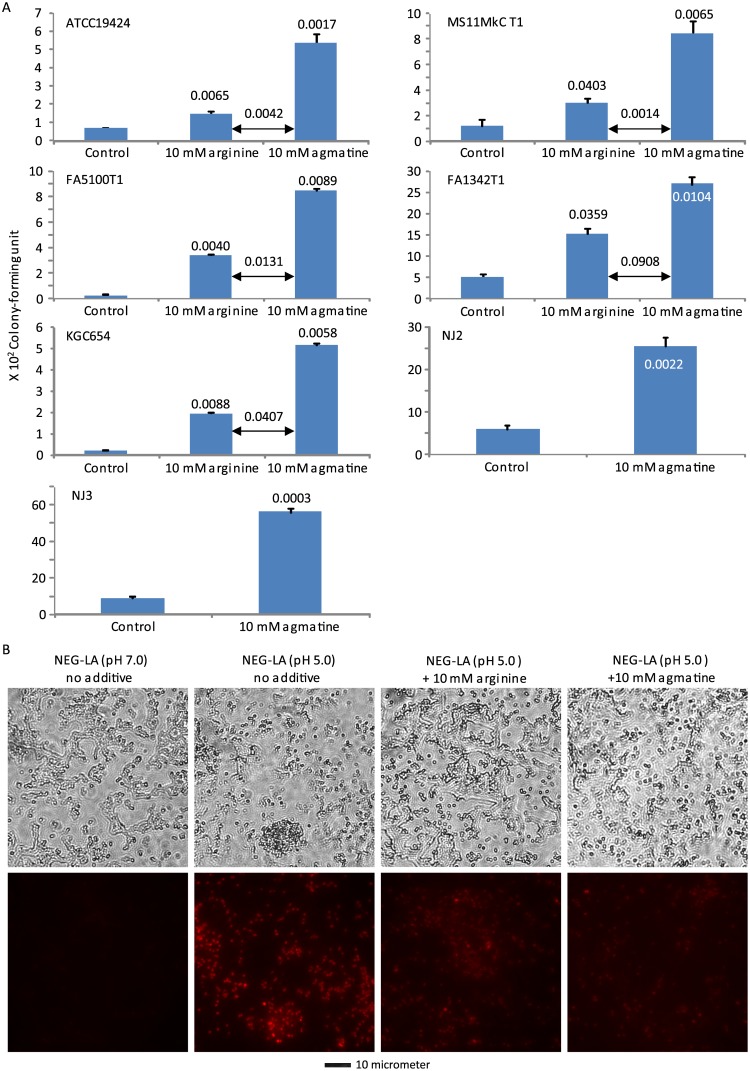
Tramine agmatine-induced acid resistance to LA-mediated killing (A) and wall/membrane permeation (B). A. Killing assays were performed as described in Fig 3 legend. B. PI staining was performed as described in Fig 3 legend. Data represent averages ± standard deviations of triplicate experiments. Numbers above the above or inside the bars were *p-*values (one-tailed *t* tests) for difference in surviving bacteria between samples treated with the control NEG-LA (pH 5.0) and those treated with NEG-LA supplemented with 10 mM arginine or 10 mM agmatine. Number of input bacteria per assay were 15,000–65,000 colony-forming units. *p-*values for differences between 10 mM arginine and 10 mM agamatine were also shown. B. Treatment and PI staining of NJ2 were performed as described in the legend to Fig 4. Matching phase contrast images (upper) and fluorescent images (lower) are presented. A scale bar is at the bottom.

Agmatine is a triamine product of arginine decarboxylase. Interestingly, agmatine also demonstrated strong protective effects in all 7 GC strains treated with NEG-LA (pH 5.0) (Fig 5A). In all 5 strains for which both arginine and agmatine were tested, inclusion of 10 mM agmatine in the medium resulted more bacteria survival than 10 mM arginine; the difference was statistically (highly) significant for 4 of the 5 strains, and trended towards significance for the remaining 1 strain (FA1342T1). Consistent with its protective function in lactic acid-induced GC killing, agmatine also strongly reduced the entry of PI into GC (Fig 5B), with 10 mM agmatine displaying a stronger effect than 10 mM arginine in decreasing PI staining. These data suggest that agmatine is responsible for the ability of arginine to stabilize the cell wall/membrane in GC in the presence of lactic acid.

### Cadaverine and putrescine decrease lactic acid-mediated GC killing and PI entry

**Fig 6:**
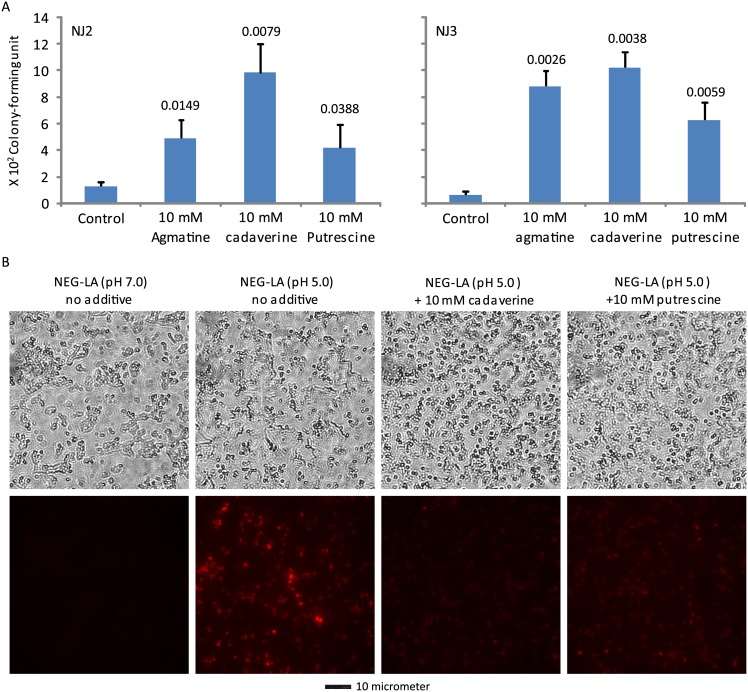
Diamines cadaverine and putrescine-induced acid resistance to lactic acid-mediated killing (A) and wall/membrane permeation (B). A. Killing assays were performed as described in Fig 3 legend. B. PI staining was performed as described in Fig 3 legend. Data represent averages ± standard deviations of triplicate experiments. Numbers above the above the bars were *p-*values (one-tailed *t* tests) for difference in surviving bacteria between samples treated with the control NEG-LA (pH 5.0) and those treated with NEG-LA supplemented with 10 mM cadaverine or 10 mM putrescine. Number of input bacteria per assay for NJ2 and NJ3 were 20,000 and 60,000 colony-forming units, respectively. B. Treatment and PI staining of NJ2 were performed as described in the legend to Fig 4. Matching phase contrast images (upper) and fluorescent images (lower) are presented. A scale bar is at the bottom.

In light of the induction of acid resistance by agmatine, we also tested the effects of two additional polyamines, cadaverine and putrescine, on lactic acid-mediated GC killing. Similar to agmatine, both cadaverine and putrescine significantly increased the survival (Fig 6A) and decreased PI entry (Fig 6B). These findings suggest that polyamines in general can decrease lactic acid-mediated GC killing by stabilizing the bacterial cell wall/membrane.

## Discussion

Lactic acid-producing bacteria create an acidic vaginal environment in most, but not all, reproductive-age women. Although previous studies have indirectly linked STI to increased vaginal pH, resulting from decreases or absence of lactobacilli [7, 15, 16], the role of lactic acid in the gonorrheal pathogenesis has not been experimentally examined to the best of our knowledge. In this report, we have demonstrated acidity-dependent GC killing by lactic acid. Thus, even though all GCL-LA contained 100 μM lactic acid, only GCL-LA at pH 4.5 or pH 4.0 were fully bactericidal, whereas the killing efficiency decreased progressively as the pH rose to 5.0 and above (Fig 1). These results are consistent with observations that neutralization of lactic acid completely abolishes the microbicidal activities towards *Chlamydia trachomatis* and HIV [23, 24]. They support the notion that the hydrogen ion but not the anion of lactic acid is responsible for killing microbes.

Many bacteria are armed with acid resistance mechanisms. In *E*. *coli*, arginine, lysine, and particularly glutamic acid can induce acid resistance. Inside the bacterial cells, these amino acids undergo decarboxylation, which consumes hydrogen ions. The decarboxylation products exit the bacterial cell in exchange for additional substrate amino acids [27, 28]. Interestingly, only arginine but not glutamic acid or lysine induced acid resistance in GC (Fig 3). Apparently, there is a correlation between the amino acid that induces resistance and the corresponding amino acid decarboxylase in GC, because only arginine decarboxylase but not glutamate decarboxylase or lysine decarboxylase is encoded by the GC genome.

Our data indicate two mechanisms are involved in GC killing by lactic acid-dissociated hydrogen ions. First, staining of acid-treated GC with the otherwise membrane-impermeable PI indicates that lactic acid causes loss of bacterial cell wall/membrane integrity. Acid-induced cell wall/membrane disruption has been demonstrated for other bacteria [29, 30]. We have not examined how lactic acid compromises the gonococcal cell wall/membrane integrity. However, previous studies have shown membrane protein expression profile changes in GC cultured in medium acidified with HCl [22, 31]. How those protein expression profile changes affect the wall/membrane integrity and bacterial survival have yet to be determined.

The ability of arginine decarboxylase, an enzyme located in the bacterial cytoplasm, to rescue GC from lactic acid-generated low pH suggests hydrogen ion-mediated inactivation of intracellular molecules as the second mechanism for lactic acid-induced GC killing. It is generally believed that arginine decarboxylase confers acid resistance by consuming hydrogen ions while removing the carboxyl group from arginine to produce agmatine and H_2_O [27, 32–36]. Interestingly, we noticed that at concentrations above 5 mM, arginine also reverses acidity-induced PI entry into GC, whereas 3 mM arginine does not appear to have such an effect (Fig 4). These observations indicate that, whereas at low concentrations, arginine induces acid resistance mainly by consuming hydrogen ions, at higher concentrations, arginine induces acid resistance by both decreasing intracellular hydrogen ions and lessening acidity-induced cell wall/membrane disruption.

In addition to arginine, its decarboxylated derivative agmatine also induces acid resistance (Figs 5 and 6). The gonococcal genome encodes an arginine-agmatine antiporter. Since the antiporter couples the exit of agmatine with the entry of arginine, agmatine may cooperate with arginine in mediating acid resistance. However, our demonstration of agmatine-induced acid resistance was made in defined medium without exogenous arginine. PI staining data indicate that inhibition of gonococcal wall/membrane disruption is the underlying mechanism for agmatine-induced, arginine-independent acid resistance. We further suggest that agmatine is partly responsible for mediating the protective effect of high arginine concentrations on the gonococcal wall/membrane integrity.

The induction of lactic acid resistance by arginine has a practical implication for gonorrheal pathogenesis because of a high level (7.3 ± 1.5 mM) of arginine in the seminal plasma [25]. In our experiments, 3–10 mM arginine increased resistance in all the 10 GC strains (Fig 3). After coitus, the concentration of seminal arginine is expected to decrease only slightly considering the volumes of ejaculates of men [37] and volumes of cervicovaginal fluids of women [38]. Thus, it is highly probable that the level of arginine in semen is high enough to promote GC survival and colonization in the vagina.

We have shown that agmatine, cadaverine and putrescine all protect GC from acid assault by stabilizing the bacterial cell wall/membrane (Figs 5B and 6B). Interestingly, previous studies have shown that polyamines including cadaverine and putrescine protect GC against human antimicrobial peptides that target lipid membranes [39]. Therefore, it is likely that other polyamines exert protective effects on acid-mediated GC killing, similar to the three polyamines that we have examined here.

Like arginine-induced acid resistance, polyamine-induced resistance also has practical implications for the pathogenesis of gonorrhea. First, there are various levels of polyamines in semen [40–42]. Thus, similar to seminal arginine, seminal polyamines may protect GC in the vagina. Second, cervicovaginal fluids also contain polyamines; bacterial vaginosis-associated microbes produce particularly high levels of diamines including cadaverine and putrescine [43–47]. Therefore, microbe-derived polyamines may increase the risks of gonorrhea by protecting the pathogen in women with mild bacterial vaginosis that still retain a relatively low vaginal pH, and by conferring resistance to antimicrobial peptides [39].

In summary, we have demonstrated lactic acid-mediated, low pH-dependent GC killing, and arginine- and polyamine-induced acid resistance. These observations are consistent with previous findings of increased risks of gonorrhea in women with bacterial vaginosis characterized by increased vaginal pH and high abundance of polyamines including cadaverine and putrescine. The revelation of arginine-induced acid resistance suggests that semen, with a basic pH and rich in arginine, protects GC through both neutralization-dependent and independent mechanisms. Likewise, the demonstration of polyamine-induced acid resistance indicates that polyamines generated by bacterial vaginosis-associated organisms can increase gonorrheal risks before the vaginal pH is raised significantly. Creation and maintenance of a sustainable acidic pH and means to decrease arginine and polyamine levels should aid in protection against GC in women and their male sexual partners in turn.
